# Shaped graft for aneurysmal bone cyst of upper limb bones

**DOI:** 10.1007/s11751-017-0291-9

**Published:** 2017-07-15

**Authors:** Mohamed F. Mostafa, Yasser Y. Abed, Sallam I. Fawzy

**Affiliations:** Orthopedic Oncology Unit, Department of Orthopedic Surgery, Mansoura University Hospital, 36 Al-Gomhoria Street, P.O. Box 35516, Mansoura, Egypt

**Keywords:** Aneurysmal, Bone cyst, Shaped graft

## Abstract

The optimal treatment of aneurysmal bone cyst remains challenging. The aim of this prospective study was to evaluate the results of using bone grafts shaped to the defects caused by aneurysmal bone cysts of upper limb bones. Fifteen patients (12 males and 3 females) with an average age of 12 years (range 6–16 years) were treated for aneurysmal bone cysts of upper limb bones by intralesional resection, argon beam coagulation and shaped bone graft. The grafts were harvested from 14 patients (11 fibulas and 3 iliac bones) and from the mother of one patient (proximal fibula). Osteosynthesis was required to stabilize the graft in four cases. The modified Enneking’s scoring system was used for functional evaluation. One patient developed partial recurrence at 6 months and required reoperation. Superficial wound infection was encountered in one patient. Shortening of the humeral segment was seen in two patients (1 and 1.5 cm) but without angular deformity. After a mean follow-up of 45 months (range 24–68 months), the mean functional score was 97.3%. This technique proved to be reliable in obtaining a well reconstructed and growing bone with no or minimal deformity and good function.

## Introduction

Aneurysmal bone cyst (ABC) is an uncommon benign tumor-like lesion of unknown origin that may present a diagnostic and therapeutic dilemma [1]. There is controversy as to whether it is a distinct radiological and pathological entity or a pathophysiological change superimposed on a pre-existing lesion [2]. The original suggestion by Lichtenstein [3] favoring a local circulatory disturbance leading to the blow-out expansion of the bone is still popular. This suggestion is further emphasized by Mirra [4] who noted that the lesion is probably a periosteal to intraosseous arteriovenous malformation. The identification of a consistent *t* (16; 17) chromosomal translocation in primary cases suggests a de novo tumor [5].

Lack of understanding about its origin and growth makes treatment empirical. The most common treatment has been intralesional excision and bone grafting with a substantial rate of recurrence ranging from 10 to 44% [6–8]. Abrasions of all surfaces using a high-speed burr and local adjuvant such as phenol, liquid nitrogen or polymethylmethacrylate (PMMA) have been tried to lower the rate of recurrence. However, the use of these adjuvants is much controversial because firm evidence that they are effective is lacking and their use entails considerable risk. Argon beam coagulation has been used as an adjuvant avoiding complications of other adjuvants [9–11].

The commonly used filling materials such as autogenous cancellous or corticocancellous bone graft, allogenic freeze-dried cancellous and cortical bone graft, PMMA and bone substitutes usually take the shape of the lesion resulting in a deformed bone with the possible limitation of function especially in the upper limb [12–14]. The current prospective study was conducted to evaluate the results of using bone grafts shaped to the original bone after extended curettage and argon beam coagulation of ABC in upper limb bones.

## Materials and methods

Between May 2005 and September 2011, 15 patients with ABCs of the upper limb bones were selected and treated at Orthopaedic Oncology Unit, Mansoura University Hospital, Egypt. For inclusion in the study which was approved by the institutional ethical research committee, all patients were briefed about the planned procedure and its possible complications and had to declare an informed consent to participate. Patients with acute pathological fracture through the cyst or secondary aneurysmal bone cyst were excluded from the study. Only patients with primary ABC of upper limb bones were included. There were 12 males and three females with an average age of 12 years (range 6–16 years) at time of surgery (Table 1). The diagnosis had been made on radiological and histological examination. Plain radiographs were performed for all cases to reveal the expanded multilocular lytic lesions. Computed tomography (CT) scan was done in 10 cases to evaluate the lesion for subtle cortical destruction or fracture. Additionally, magnetic resonance imaging (MRI) was performed in eight cases to identify the characteristic double density fluid levels and septations as well as the extension to the epiphysis. Tissue for histological examination was obtained by trephine biopsy. Two patients with recurrent lesions were biopsied preoperatively to confirm diagnosis. The presence of blood-filled cystic spaces separated by fibrous septa (membranes) of mononuclear stromal cells containing scattered multinucleated giant cells and less commonly reactive bone was suggestive of diagnosis.

**Table 1:** Details and results in 15 patients with aneurysmal bone cysts of upper limb bones

Case no.	Age	Gender	Location	Stage	Size (cm^3^)	Follow-up (month)	Time to consolidation (week)	Score^a^	Complications
1	6	Female	distal Radius	Stage 3	48	68	16	97	No complication
2	12	Male	Distal humerus	Stage 3	60	60	20	100	No complication
3	15	Male	Distal humerus	Stage 2	30	55	16	100	No complication
4	16	Female	Distal humerus	Stage 3	60	52	18	100	No complication
5	8	Male	Proximal humerus	Stage 3	66	50	18	97	Partial recurrence
6	10	Male	Proximal humerus	Stage 2	60	54	12	97	Superficial infection
7	11	Male	Proximal humerus	Stage 2	40	45	16	100	No complication
8	16	Male	Proximal humerus	Stage 3	150	50	22	90	Shortening of humerus
9	14	Male	Shaft humerus	Stage 2	72	41	20	100	No complication
10	7	Male	Distal radius	Stage 2	30	40	12	97	No complication
11	11	Male	Shaft humerus	Stage 2	99	39	14	100	No complication
12	13	Male	Proximal humerus	Stage 3	105	35	20	97	No complication
13	14	Male	Proximal humerus	Stage 2	60	29	16	97	No complication
14	15	Female	Proximal humerus	Stage 3	240	25	22	87	Shortening of humerus
15	10	Male	Proximal humerus	Stage 2	52	24	14	100	No complication

^a^Enneking scoring system (rating percentage of normal) [16]

**Fig. 1:**
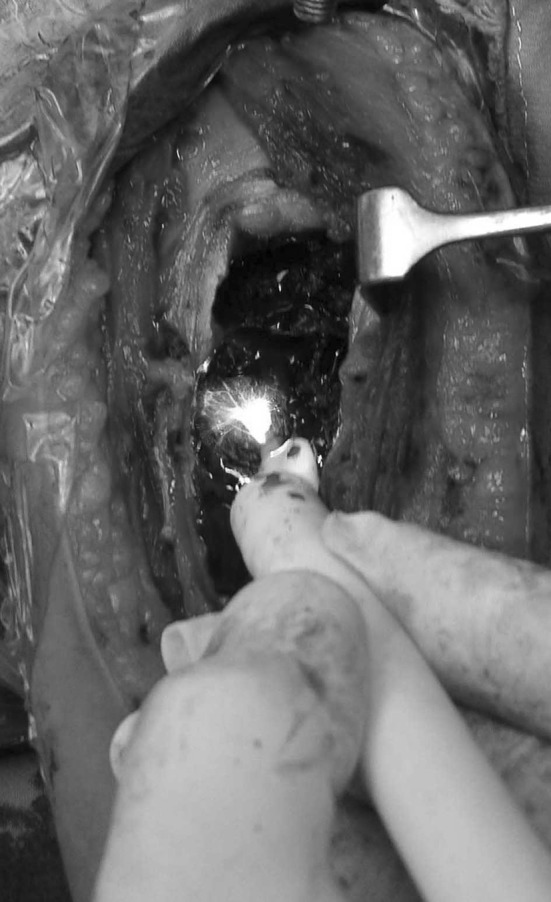
An intraoperative photograph showing the argon beam coagulator wand used as a paint brush with eschar formation throughout the entire inner wall

The lesion was located in proximal humerus in eight patients, distal humerus in three, shaft humerus in two and distal radius in two. The approximate volume of the cyst was calculated using plain radiograph and more accurately CT scan by multiplying the maximum length and breadth in anteroposterior projection and the depth in lateral projection. Staging was accomplished using the criteria defined by Enneking [15]. Operative treatment consisted of a wide window, extended curettage, argon beam coagulation and graft insertion. Curved small curettes were helpful to reach small pockets and slits especially near the diaphyseal side. Care was taken while curetting the lesion close to the physis. A power burr was used to extend the margin of excision and to open the medulla in some cases but was avoided at the broached areas of physis. Argon beam coagulator (Birtcher 6000 Electrosurgical Generator + Argon Beam Coagulator, Irvine California, USA) was used like a paint brush throughout the entire inner wall and at a minimum near the physis. This produced a thin layer of eschar due to deposition of black carbonized debris (Fig. 1). Simultaneous irrigation and suction was helpful to clear debris and eschar, prevents contamination and allows for improved visualization. The sequence of curettage, argon beam coagulation and lavage was repeated three to four times before application of the graft.

**Fig. 2:**
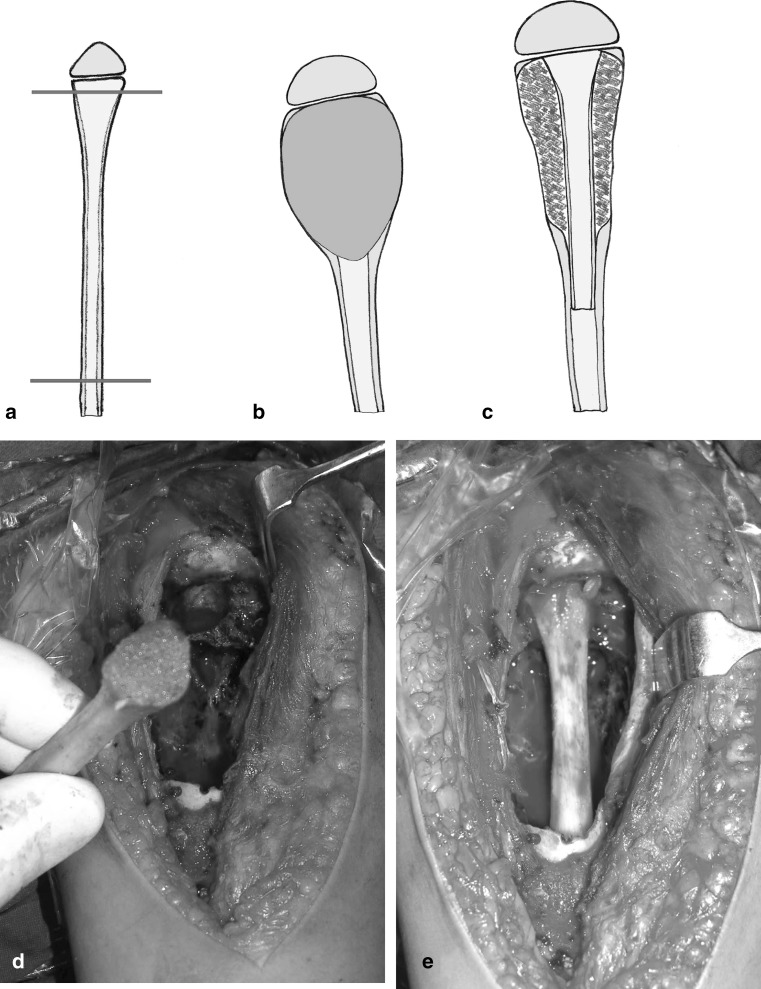
**a**–**c** Diagrams showing the selected part of the proximal fibula placed after extended curettage of a proximal humeral lesion, the expanded outer cortex was gently collapsed and composite bone substitute was used to fill the gaps. **d** The proximal fibular graft provides a smooth cancellous surface. **e** After placement of the graft with the cancellous surface opposite the broached area of the physis

**Fig. 3:**
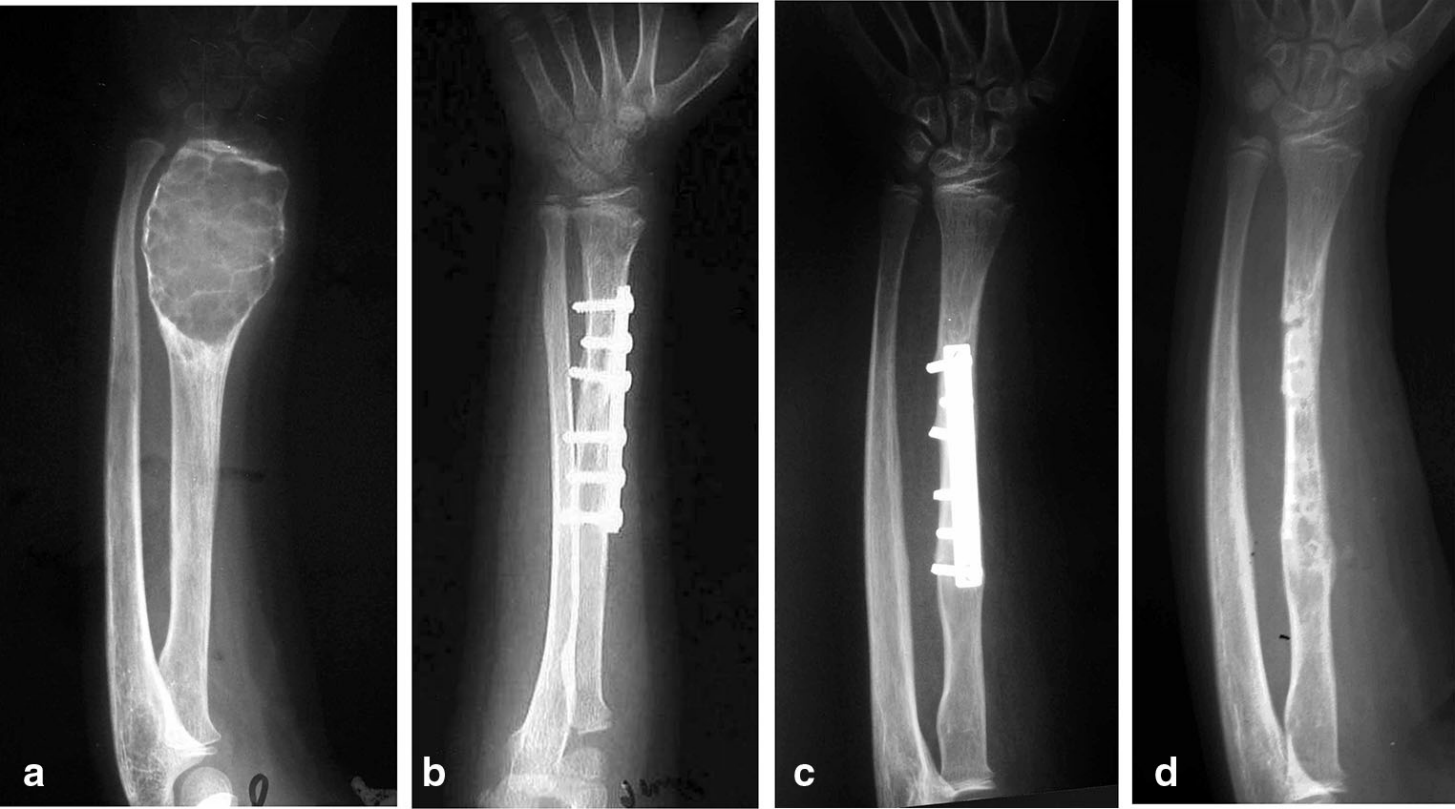
**a** Anteroposterior radiograph of a 6-year-old girl with ABC destructing the distal one-third of left radius (Case 1). **b** Proximal fibular graft was harvested from her mother, shaped and stabilized with plate and screws. **c** Follow-up radiograph showing continued growth of the graft and maintained open physis. **d** 5 years after surgery with continuing growth and no deformity

**Fig. 4:**
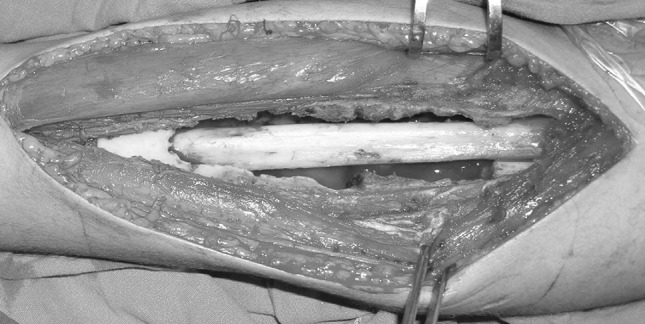
Photograph showing fibular shaft graft placed intramedullary in humeral diaphyseal lesion

**Fig. 5:**
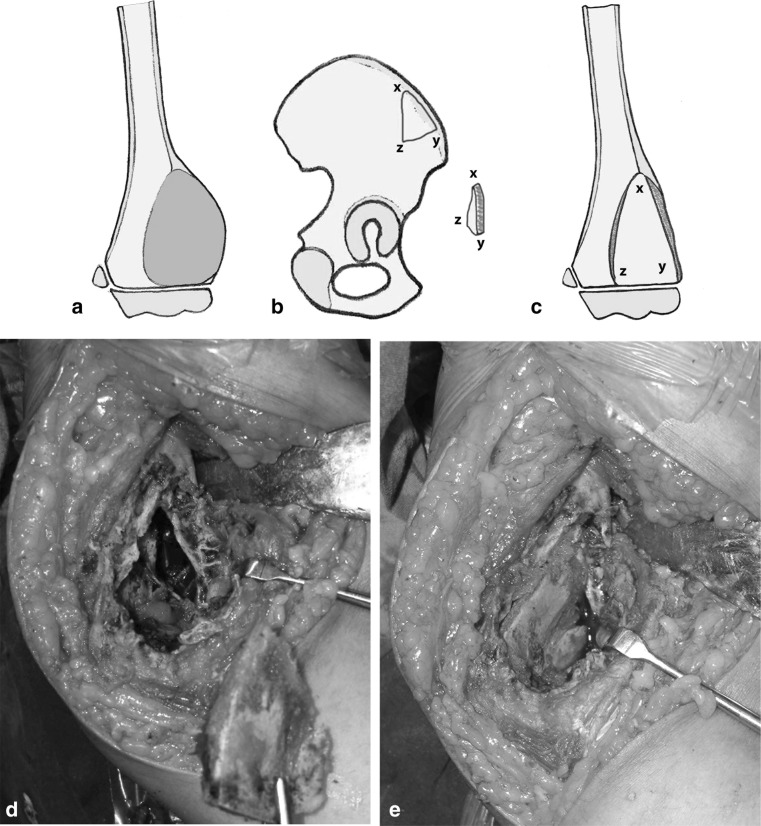
**a**–**c** Diagrams of an eccentric distal humeral ABC, the planned area of iliac bone graft and placement of the graft with the thick portion (*x*–*y*) laterally and the thin apex (*z*) centrally toward the area where coronoid and olecranon fossae met. **d** An intraoperative photograph showing the iliac bone graft shaped to the defect in the medial humeral condyle (Case 4). **e** The graft in place

After careful measuring the defect preoperatively and intra-operatively, the graft was harvested from the patient (proximal fibula = 9, shaft fibula = 2, iliac bone = 3) and from the patient’s mother (proximal fibula = 1). Proximal fibular grafts taken from the patients provided a smooth cancellous surface for the exposed areas of proximal humeral physis (Fig. 2). The proximal fibular graft harvested from the mother of a 6-year-old girl (case 1) was cleaned from soft tissue, washed thoroughly with saline and reshaped before placement in the defect caused by ABC of the distal third radius. In this case, the proximal smooth cancellous surface was applied against the growth plate and the distal cortical part was internally fixed to the radius with plate and screws (Fig. 3). The fibular shaft graft was inserted retrograde into the proximal humerus then impacted back into the distal part in cases with diaphyseal lesion (Fig. 4). One of the two patients with diaphyseal ABCs developed intraoperative humeral shaft fracture during graft insertion and required internal fixation with plate and screws. A composite of synthetic bone substitute (ceraform; calcium phosphate hydroxyapatite 65% and tricalcium phosphate 35%, Teknimed S.A. France) and bone marrow aspirate was used to fill the remaining gaps around the graft. The distal humeral lesions were eccentric involving medial condyle in two patients and lateral condyle in one patient. These patients were older enough to obtain autogenous bone graft from their iliac bone. The graft was shaped and placed with the thick portion of the iliac crest at the periphery representing the medial or lateral column and the thin portion centrally toward the thin area of distal humerus where olecranon and coronoid fossae meet (Fig. 5). Smooth Kirschner wires were used to stabilize the graft in two patients. The expanded outer shell was collapsed manually and gently over the graft.

Patients with humeral lesions were instructed to keep the limb in arm sling till suture removal then started early passive range of motion (ROM) of shoulder, elbow and wrist. Isometric strengthening exercises and active ROM were postponed till complete healing of the lesion. For cases with distal radial lesions, a below elbow plaster cast was applied for 5–6 weeks. All patients were allowed to start finger movement early after surgery guided by pain. The mean duration of follow-up was 45 months (range 24–68 months). Patients were evaluated radiologically by plain radiographs every 4–6 weeks for progression of healing, local recurrence and deformity resulting from partial fusion of the epiphysis. After radiographic healing, patients were assessed clinically every 3 months for the first year and every 6 months during the 2nd year then yearly thereafter. Clinical assessment was done for pain, deformity, limitation of joint movement and complications at the donor site. The modified Enneking scoring system [16] was used for functional evaluation at the time of final follow-up.

## Results

The main presenting symptoms were pain and discomfort associated with swelling in 11 patients. The remaining four patients had repeated pathological fractures of a stage 3 proximal humeral lesions. One of them had significant varus deformity with limitation of abduction and was corrected at the time of surgery. There were eight lesions stage 2 and seven stage 3 with a mean size of 78 cm^3^ (range 30–240 cm^3^).

All lesions healed uneventfully after a mean time of 17 weeks (range 12–22 weeks). Only one patient (case 5) developed partial recurrence at 6 months that required reoperation. The collapsed outer shell disappeared with the progress of healing. The rapid healing of the lesion was closely related to the young age of patients, the small size and the less aggressive (stage 2) lesions. Most of patients (83.3%) with open physis and juxta-physeal lesions had continued growth in length and width as evidenced by absence of deformity or shortening and maintained open physis. The patient who received fibular graft from her mother showed complete incorporation and remodeling of the graft with continued growth of the radius and no deformity. Only two patients (16.7%) developed shortening of the humeral segment (1 and 1.5 cm) due to premature fusion of the proximal humeral epiphysis but without angular deformity. These two patients had large size lesions (mean 195 cm^3^) of the proximal humerus with marked broaching of the physis.

There were no cases of deep infection, nerve deficit or pathological fracture. Superficial infection of the operative wound was seen in one patient and was controlled with repeated dressing and systemic antibiotics. The mean functional score (rating percentage of normal) at treatment completion was 97.3% (range 87–100%). There were no complications related to the donor site. Subperiosteal harvesting of the fibular graft allowed regeneration of a new fibula. Postoperative pain, abductor dysfunction and limping after harvesting iliac bone graft could be avoided by proper soft tissue repair.

## Discussion

ABC is one of the aggressive benign bone tumors. Despite being described for more than 60 years back, still there is controversy about its nature, pathophysiology and optimal treatment. Appropriate treatment can be made only after the exclusion of an underlying lesion particularly giant cell tumor [17]. The use of physical adjuvants such as phenol, liquid nitrogen and polymethylmethacrylate (PMMA) has been advocated to extend the surgical margin. These adjuvants produce chemical or thermal necrosis and microvascular damage to the walls of the physically excised cyst aiming to decrease the chance of recurrence. However, phenol and liquid nitrogen could penetrate tissues making the neurovascular structures at high risk. Also, liquid nitrogen can make the bone more brittle and increase the risk of fracture [6].

Lower recurrence rates can also be achieved by marginal or wide resection, but this entails loss of the supporting function of bone and the need for reconstructive surgery [18, 19]. Furthermore, others do not believe it is necessary to perform wide excision to eradicate the disease [20, 21]. In the current study, the cyst walls were not excised, instead allowed to collapse over the implanted graft to provide the new periosteum for growth in width. Local recurrence was seen only in one patient (7%) and was partial at the periphery of a large stage 3 proximal humeral lesion. In agreement with Gitelis and McDonald [22], local recurrence depends mainly on the adequacy of the tumor removal rather than the type of adjuvant used. This is achieved by making a large window to expose the whole cavity, using different sizes curettes, good visualization of the inner walls with the help of a small light source fixed to the suction nozzle and the use of power burr. The use of a high-speed burr has been suggested to reduce the rate of recurrence [23]. However, Lin et al. [21] detected no difference in recurrence rate with or without the use of high-speed burr. On the other hand, high-speed burr could not be used at the open physis, as this could destroy the growing cells and cause cessation of growth. It is believed that meticulous curettage with small curettes is preferred for the physeal borders.

Argon beam coagulator is a monopolar coagulator which utilizes a beam of argon gas to deliver a radiofrequency electric current to the tissues. The jet of gas blows blood and debris away and allows the inner surface of the cavity to be covered with a thin layer of eschar causing coagulation of blood in the lumen of small vessels and capillaries. The electrical current flows through the gas in arcs that are distributed uniformly across the tissue in depth and area, with tissue effects depending on the power setting and duration of application. The temperature of the tissue reaches a maximum of 205 °C when the spray passes over the tissues. The residual tumor cells in the cavity are destroyed by this thermal effect. Compared with other adjuvant treatments, the limited beam length and area and the directional nature of the beam make this technique simple to use, precise and safe if the nearby neurovascular bundles are protected [10]. Cummings et al. [9] in a preliminary study found that argon beam coagulation after curettage provided improved local control of ABCs compared with curettage without adjuvant or with phenol and not associated with an increase in the operative complications. In the current study, argon beam coagulation was used as an adjuvant in all cases and there were no difficulties with its use or postoperative complications related to it.

It is noted that the final shape of the new bone will correspond to the one developed by the cyst at the time of surgery. Stuffing the expanded lesion with the commonly used filling materials such as autogenous cancellous or corticocancellous bone grafts, allogenic cancellous or cortical bone graft or bone substitutes including PMMA usually result in a bulky and deformed bone with possible premature fusion of the nearby epiphysis and shortening [24]. In the present cases, bone graft was intra-operatively shaped with the smooth cancellous surface placed opposite the exposed area of the physis allowing a regular enchondral ossification. This was evidenced by the maintained open physis, continued growth in length and absence of deformity. Furthermore, the expanded outer cortex was collapsed gently providing the graft its new periosteum for increase in width.

Autogenous bone graft remains the gold standard as a filler for cavity defects. It demonstrates a high degree of osteoinduction from surface osteoblasts in the host bone, circulating osteoprogenitor cells and donor cells that survive transplantation. In addition fresh transplanted autogenous bone exhibits no immune response and undergoes rapid revascularization. However, autogenous bone graft is sometimes not available in sufficient quantity and its harvest has the potential complications of pain, blood loss, increased operative time, infection and donor site instability [25]. No perioperative or postoperative complications were encountered with harvesting bone graft in the current study. The morbidity could be avoided or minimized if the graft harvesting technique is properly planned and performed. In our study, one young girl received fibular strut graft from her mother and showed complete healing without any evidence of immunological reaction or rejection. The graft was transplanted fresh without processing other than removal of soft tissues and washing with saline. In agreement with Weber et al. [26], syngenesioplastic graft obtained from immediate relatives of the patient could be a reasonable alternative to autogenous bone graft for small children. The main weakness of this study was the limited sample size. Despite this recurrence rate, complications and functional results at the final follow-up were comparable to that of other studies using different methods of treatment and adjuvants (Table 2).

**Table 2:** Reported studies using different adjuvant agents compared with the current study

Study	Number of patients	Treatment	Adjuvant	Number of recurrence (%)	Mean functional score^a^	Complications
Ozaki et al. [12]	14	Curettage	PMMA in 5	2 (14%)	NS^b^	1 fracture 3 joint stiffness
Dormans et al. [27]	45	Curettage	Cauterization Phenol Hydrogen peroxide	8 (18%)	NS	–
Basarir et al. [28]	56	Curettage Resection	Cauterization Embolization	9 (16%)	NS	4 deformities 2 limb inequalities 1 infection
Peeters et al. [29]	80	Curettage Bone graft in 73	Liquid nitrogen	4 (5%)	97.3%	1 postoperative fracture 1 wound infection 3 transient nerve palsy
Cumming et al. [9]	29	Curettage Resection	Argon beam coagulation in 17	4 (14%)	NS	1 physeal arrest
Current study	15	Curettage Bone graft	Argon beam coagulation	1 (7%)	97%	1 superficial wound infection 2 shortening of humeral segment

^a^Modified Enneking scoring system [16]

^b^ *NS* not stated

ABC remains an enigma not only regarding origin but also diagnosis and optimal treatment. The use of a shaped strut bone graft after meticulous curettage and argon beam coagulation is a reliable technique to obtain a well reconstructed and growing bone with minimal or no deformity and good function for ABCs of the upper limb bones. It is not necessary to perform wide excision to eradicate the disease. Great care should be exercised while removing the tumor near the physeal cartilage to avoid injury to the growing cells and premature epiphyseal fusion. Considering the limited quantity and the morbidities of harvesting autogenous bone graft, the author is looking forward for a shaped bone substitute that can be mixed with bone marrow as a source of osteogenic cells to aid in healing of such lesions.
